# A strategy for the identification of patterns in the biosynthesis of nonribosomal peptides by Betaproteobacteria species

**DOI:** 10.1038/s41598-017-11314-w

**Published:** 2017-09-04

**Authors:** João Luiz Baldim, Bruna Lidiane da Silva, Daniela Aparecida Chagas-Paula, João Henrique G. Lago, Marisi G. Soares

**Affiliations:** 1 Institute of Chemistry, Federal University of Alfenas, Alfenas, MG 37130-000 Brazil; 2 Centre of Human and Natural Sciences, Federal University of ABC, Santo André, SP 09210-580 Brazil

## Abstract

Nonribosomal peptides have an important pharmacological role due to their extensive biological properties. The singularities in the biosynthesis of these natural products allowed the development of genome-mining strategies which associate them to their original biosynthetic gene clusters. Generally, these compounds present complex architectures that make their identification difficult. Based on these evidences, genomes from species of the class Betaproteobacteria were studied with the purpose of finding biosynthetic similarities ﻿among them﻿. These organisms were applied as templates due to their large number of biosynthetic gene clusters and the natural products isolated from them. The strategy for Rapid Identification of Nonribosomal Peptides Portions (RINPEP) proposed in this work was built by reorganizing the data obtained from antiSMASH and NCBI with a product-centered way. The verification steps of RINPEP comprehended the fragments of existent compounds and predictions obtained *in silico* with the purpose of finding common subunits expressed by different genomic sequences. The results of this strategy revealed patterns in a global overview of the biosynthesis of nonribosomal peptides by Betaproteobacteria.

## Introduction

Microorganisms produce a large variety of Natural Products (NPs) known for their biological properties^1–3^. These chemical entities belong to a wide range of classes that exhibit anticancer, antibiotic, immunosuppressant, cytostatic, and several other essential effects for maintaining the current level of human health^2–4^.

Some of these NPs are from one of the most important classes of drugs in clinical use as the nonribosomal peptides^2^. They are designed for a specific target, since they were optimized for millions of years due to evolutionary pressure and biological purposes^5^. Nonribosomal peptides are biosynthesized by Nonribosomal Peptide Synthetases (NRPS) that are groups of specialized and modular enzymes functionalized to activate, to bind, and to incorporate building blocks into the final polypeptide structure, resulting in a huge diversity and complexity of compounds^6, 7^. The biological properties of these nonribosomal peptides are resultant of some structural modifications, such as cyclization of opened chain amino acids and/or posttranslational modifications which make it challenging to recognize patterns in their biosynthesis^8–10^. The presence of specialized domains related to these modifications are often associated to the amino acids serine, threonine, and cysteine producing respectively, oxazole, methyloxazole and thiazole rings^8, 10–12^. Eventually, the presence of these heterocycles in the nonribosomal peptide structure changes their typical chemical shift in NMR analysis, suggesting that they could be cyclized instead of an opened side chain. However, they could be identified by the occurrence of sp^2^ carbons instead of sp^3^ carbons and the lack of amide protons related to these three heterocyclic subunits^10, 12^.

In this context, there are different approaches based on genome-mining strategies which provided methods for the discovery of new chemical entities (NCEs) from natural sources^13, 14^. Predominantly‚ these methods are based on genomic analysis designed to the identification of essential genes for the biosynthesis of NPs. These genes are co-located on the microorganism’s genomes and operate collectively as biosynthetic gene clusters (BGCs)^15^.

Genome-mining methods can be applied to nonribosomal peptides due to the characteristics of their biosynthesis, that is based on monomers being incorporated one by one forming the polypeptide structure^16, 17^. The biosynthetic core of nonribosomal peptides contains at least three domains per module^7^. These NRPS domains are named adenylation, A domains (associated with chain initiation, responsible for substrate specificity and activation); condensation, C domains (associated with chain elongation, that catalyzes the peptide bond formation between a new substrate and the peptide chain), and thioesterification, Te domains (associated with chain termination, responsible for the peptide releasing)^7, 16, 17^. The number of monomers in the final structure corresponds to the number of modules in the NRPS^10, 18^. It is important to highlight that the biosynthetic domains of nonribosomal peptides also allow some specific variations accepting different amino acids, increasing the complexity of their final structures^6, 19, 20^. This event is described as substrate promiscuity or flexibility, resultant of responses to chemical or biological stress^21^.

The evolution of bioinformatics tools and the increasing amount of fully sequenced genomes made it possible to explore the hidden potential of microorganisms concerning the production of secondary metabolites^15, 22, 23^. These facts have revealed a vast discovery scenario where the total number of NPs is rather smaller than the number of gene clusters, suggesting that there are plenty to be explored from studied and unstudied species^24^. The power of these computational methods also created dereplication techniques that become essential to the investigation of microbial extracts in the search of NCEs^25–30^.

The similarity of genomes in taxonomically close species result in the biosynthesis of similar compounds wherein the level of equivalence among gene clusters and compounds are directly proportional^6, 31, 32^. In this sense, species of the class Betaproteobacteria (BPB) have emerged as promising species regarding their biosynthetic potential. These species present a large number of BGCs in their genomes. Representatives of this class are Gram-negative bacteria often considered neglected producers of antibiotics, as in the case of the genera *Pantoea* sp., *Janthinobacterium* sp., and *Burkholderia* sp. However, after genome-guided strategies applied to some of these species, they afforded novel nonribosomal peptides NPs such as jagaricin, janthinocin, and a polyketide product, enacyloxin IIa, which all exhibit antibiotic properties^33^.

Another successful approach with the class BPB is the discovery of the nonribosomal peptide teixobactin^34^. This investigation is an outcome of methods applied to the investigation of the uncultured microbiome resulting in the isolation of a representative of the class BPB, *Eleftheria terrae*, and the following isolation of teixobactin from this strain. Teixobactin is an antibiotic active against Gram-positive and mycobacteria as a lipid II antagonist.

Due to these evidences, a simple strategy for a Rapid Identification of Nonribosomal Peptides Portions (RINPEP) was developed for measuring structural similarities among gene clusters products. The RINPEP dataset for BPB was built by merging the data obtained from antiSMASH^35^ results and NCBI. This strategy was built based on the existence of sites that do not allow substrate flexibility in the biosynthesis of nonribosomal peptides. Thus, in order to identify biosynthetic patterns we proposed to investigate monomers often linked by equivalent gene clusters in different species. This strategy revealed patterns in the biosynthesis of nonribosomal peptides by species of the class BPB in a product-centered approach.

## Results

### General classification of BGCs from species of the class Betaproteobacteria

**Figure 1:**
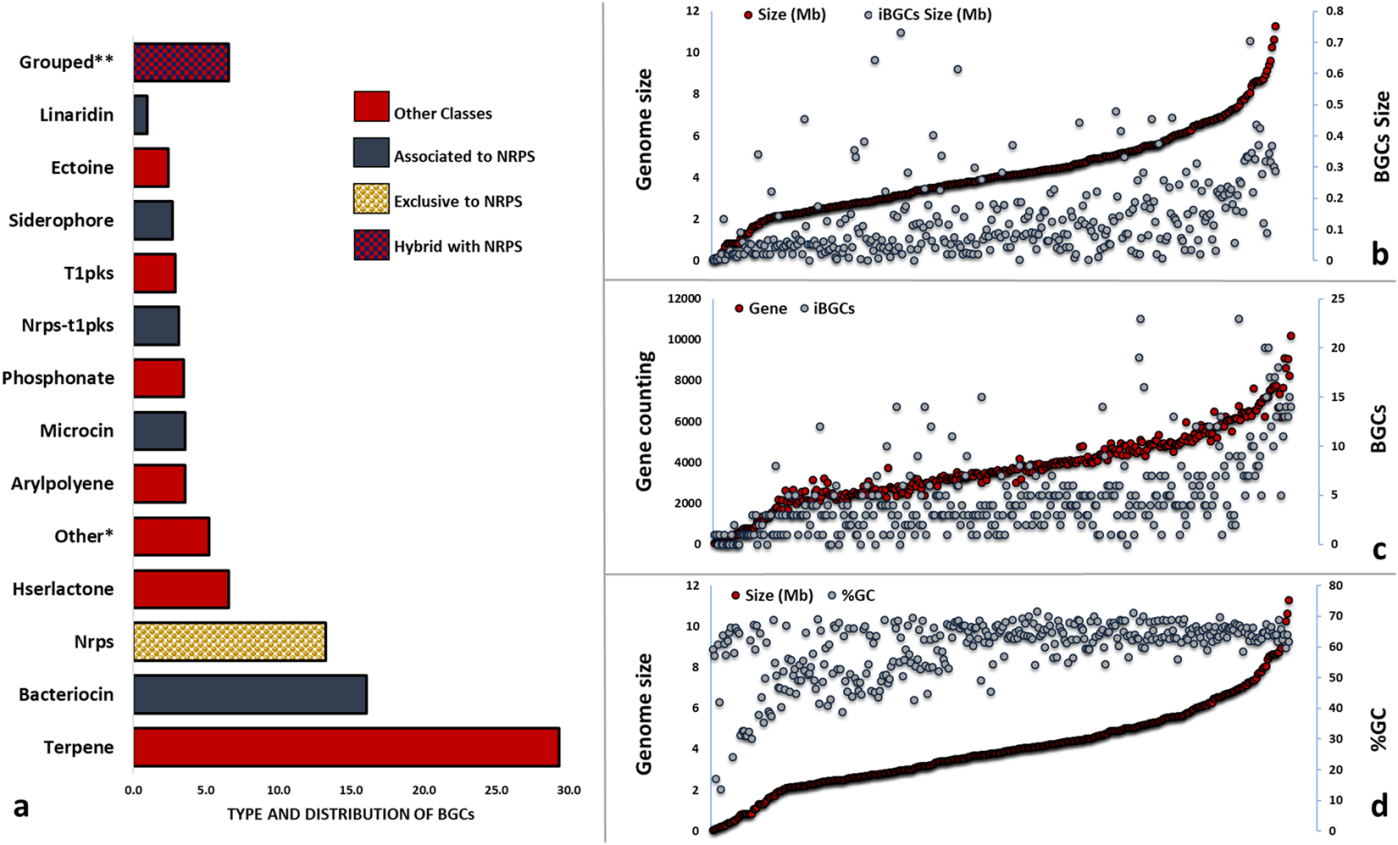
The classification of BGCs identified in genomic sequences of BPB species. (**a**) The general distribution of BGCs (in percentage). (**b**) Gene cluster size *vs*. genome size. (**c**) Gene counting *vs*. number of gene clusters. (**d**) %GC *vs*. genome size. *Other classes (not identified by antiSMASH). **Other classes identified by antiSMASH (grouped).

The results of antiSMASH afforded around 1650 BGCs from 359 genomic sequences investigated. Approximately 13.5% of these gene clusters (237 BGCs) pointed to nonribosomal peptides NPs. The results also exhibited different gene clusters classified as hybrid, such as NRPS-T1PKS, HGLKS-NRPS-T1PKS, corresponding to 28% of all results (Fig. 1a). Altogether, the sequences associated with the production of nonribosomal peptides corresponded to 40% of the gene clusters identified.

Species with larger replicons dedicate more of their genomes to the biosynthesis of NPs (Fig. 1b). The number of genes obtained from BPB genomes in NCBI is directly proportional to the number of gene clusters obtained from antiSMASH results (Fig. 1b). The genomes of BPB species exhibit an average GC-content of 65% (Fig. 1d). Genomes rich in GC are associated to evolutionary questions, that could be related to the stability of enzymes, protein structures, and improving nutrients absorption^36, 37^.

The results that pointed to the existence of hybrid gene clusters predicted subunits from two different biosynthetic pathways (besides those of NRPS-PKS) corresponded to 3.24% of all BGCs (57 gene clusters) in which 23 were related to NRPS and 34 not related to NRPS. A large percentage of these gene clusters were associated with the production of terpenes (496 BGCs, 28.23%) and bacteriocins (271 BGCs, 15.42%). Approximately 250 BGCs did not present association with any known gene cluster, suggesting that NCEs might possibly be expressed. In addition, some gene clusters presented levels of genomic identity with taxonomically distant species as in the case of Bacillibactin (from Firmicutes; identity from 8 to 30%), Tubulysin (from Myxobacteria; identity of 6%), Laspartomycin (from Actinobacteria; identity from 6 to 28%), and Fuscachelin (from Actinobacteria; identity from 10 to 30%).

### RINPEP dataset design and tests

The results obtained from the platform antiSMASH provided two important variables for the development of the RINPEP dataset: a) the predicted core structure associated to each BGC; and b) the homology with gene clusters that regulate biosynthesis of known compounds. The gene cluster 5 of *B. thailandensis* illustrate the variable b: Type: NRPS; most similar known gene cluster: Burkholdac, 100% of genes show homology, MIBiG BGC-ID: BGC0000964_c1. These two results made it possible to create variables for tracking patterns of amino acids, in which pairs of predictions (POPs) and pairs of existent compounds (PECs) were created by *in silico* fragmentation of their original structures. POPs and PECs were utilized for inferring about the distribution of patterns in the biosynthesis of nonribosomal peptides. The percentage of homology between predictions and existent compounds was investigated through dynamic network, Jaccard Index of similarity, and phylogenetic analysis. The RINPEP dataset is available as a heatmap based on POPs and genus at the Supplementary Session as Supplementary Figure 1, part 1 and 2.

In this investigation, 359 genomes were used, including drafts. The reason of using low quality genomes along with those of high quality is to be more comprehensive. Low quality genomes would result in the prediction of core structures or only in the detection of a possible peptide backbone. The information for the creation of POPs is naturally filtered, and obsolete results from draft genomes are excluded if there is no prediction. These data are only present in the final dataset whether their results mandatorily presented POPs.

The strategy followed a two-step analysis, which connected gene clusters, their respective PECs, and POPs. The two-step verification consisted of the evaluation of results from predictions in two forms -first: PECs *vs*. POPs (eg. estimates the incidence of PECs matching predictions with a cutoff of five in the number of directed edges from the main network); and -second: POPs *vs*. PECs (eg. estimates the incidence of similar POPs with levels of genomic identity between gene clusters).

A dynamic network was built with the purpose of associating BGCs by their levels of homology based on the most similar known gene cluster indicated by antiSMASH. A .csv file containing source (microorganism name) and target information (the final NP with the respective level of gene cluster similarity) was submitted to Cytoscape 3.3.0 resulting in a network for the investigation of matches between PECs *vs*. POPs.

**Figure 2:**
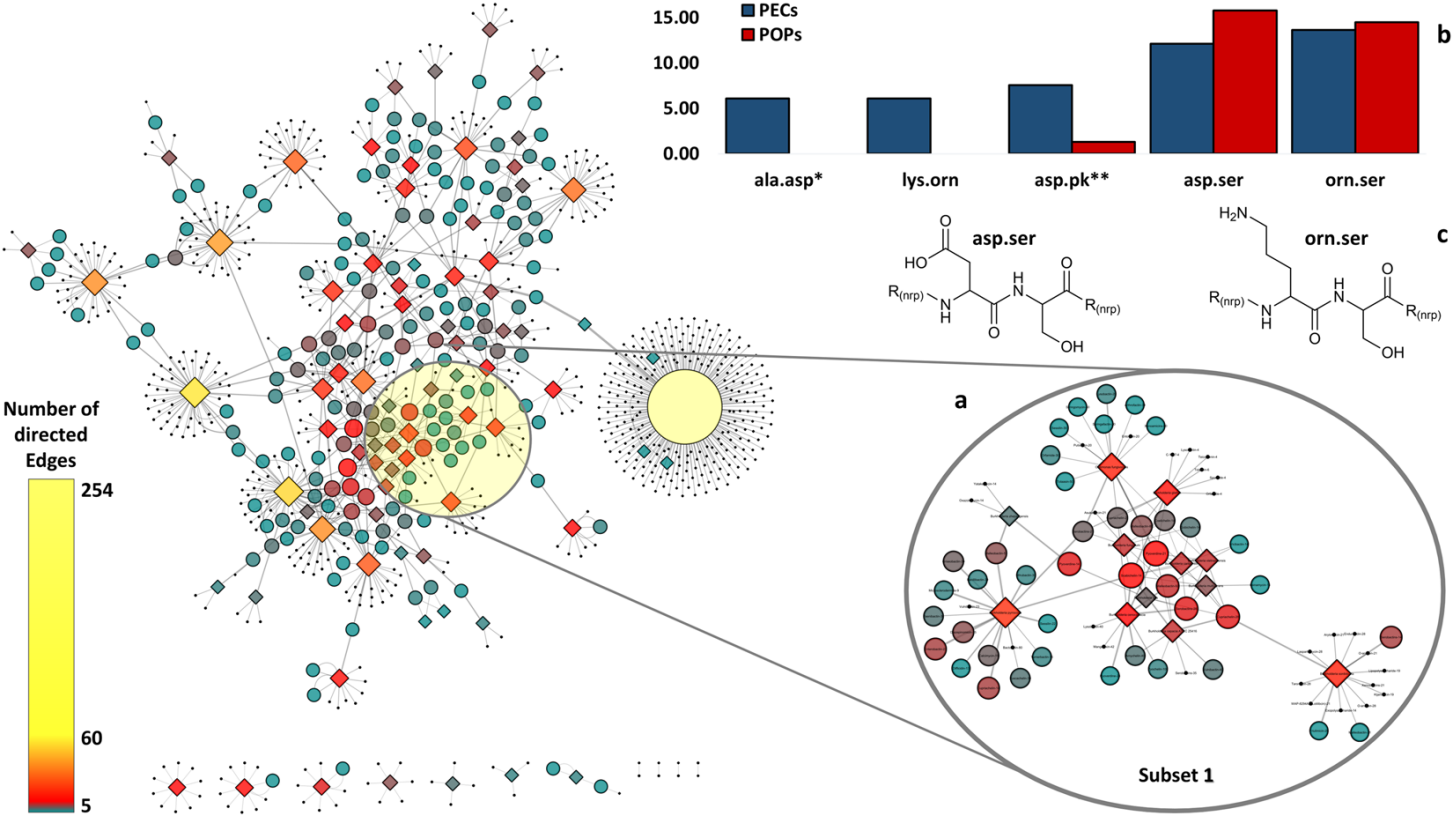
The dynamic network with microorganisms and the level of homology with existent compounds. (**a**) The subset 1 and its first neighbors (compounds) selected. (**b**) Percentage of PECs and POPs for the compounds associated with species present in the subset 1. (**c**) The pairs orn.ser and asp.ser are highlighted. The dynamic network graph was built using Cytoscape 3.3.0. The subset 1 was created excluding number of directed edges lower than 5 correlations in Cytoscape. Nodes and color scale are related to their number of directed edges. The diamond shape represents microorganisms and the circle shape represents compounds and their level of homology. The network was treated with Spring-Electric Algorithm and Allegro Layout version 2.2.1. The biggest node is related to unknown BGCs. *ala.asp and lys.orn were not predicted. **asp.pk is a hybrid portion associated to T1PKS-NRPS pathway.

The fragments of predicted core structures and existent compounds composed the dataset of POPs and PECs, respectively. A region in the main network was randomly selected and called Subset 1 (Fig. 2a). The species in this subset had their PECs and POPs deposited in a binary matrix (0 = absent, 1 = present) where the microorganisms were considered instances and the fragments, attributes. The objective of this step was the creation of a comparable dataset for PECs and POPs. The results of this analysis exhibited a high level of similarity between PECs and POPs mainly associated to the fragments orn.ser (predicted 14.47%, observed 13.64%), and asp.ser (predicted 15.79%, observed 12.12%) (Fig. 2b) which were expressed equally in both, real and predicted.

The results of antiSMASH predicted that several gene clusters presented genomic identity to those already catalogued in the platform MIBiG^38^. These results selected compounds that might be similar to the NP biosynthesized by the original gene cluster. These data made it possible to compare POPs and PECs allowing the application of the RINPEP dataset as a support for the identification of patterns in the biosynthesis of nonribosomal peptides from BPB species. This affirmation was taken based on the values of Jaccard Index (JI), further discussed ahead. It is important to highlight that the number of proteinogenic and nonproteinogenic peptides are greater than 500 different subunits^39^, creating a vast number of different building block connections. These connections could greatly complicate the recognition patterns in NPs. Thus, these biosynthetic patterns shed light on the most common building blocks used by these NRPS.

Besides, the subset 1 revealed that a proportion of gene clusters for the biosynthesis of nonribosomal peptides are hybrid. The Fig. 2 shows that POPs of this subset are also associated with type 1 polyketide synthases (T1PKS) products. These gene cluster moieties were associated with the union of aspartate and a polyketide moiety in the form of asp.pk (1.32%, Fig. 2b).

**Figure 3:**
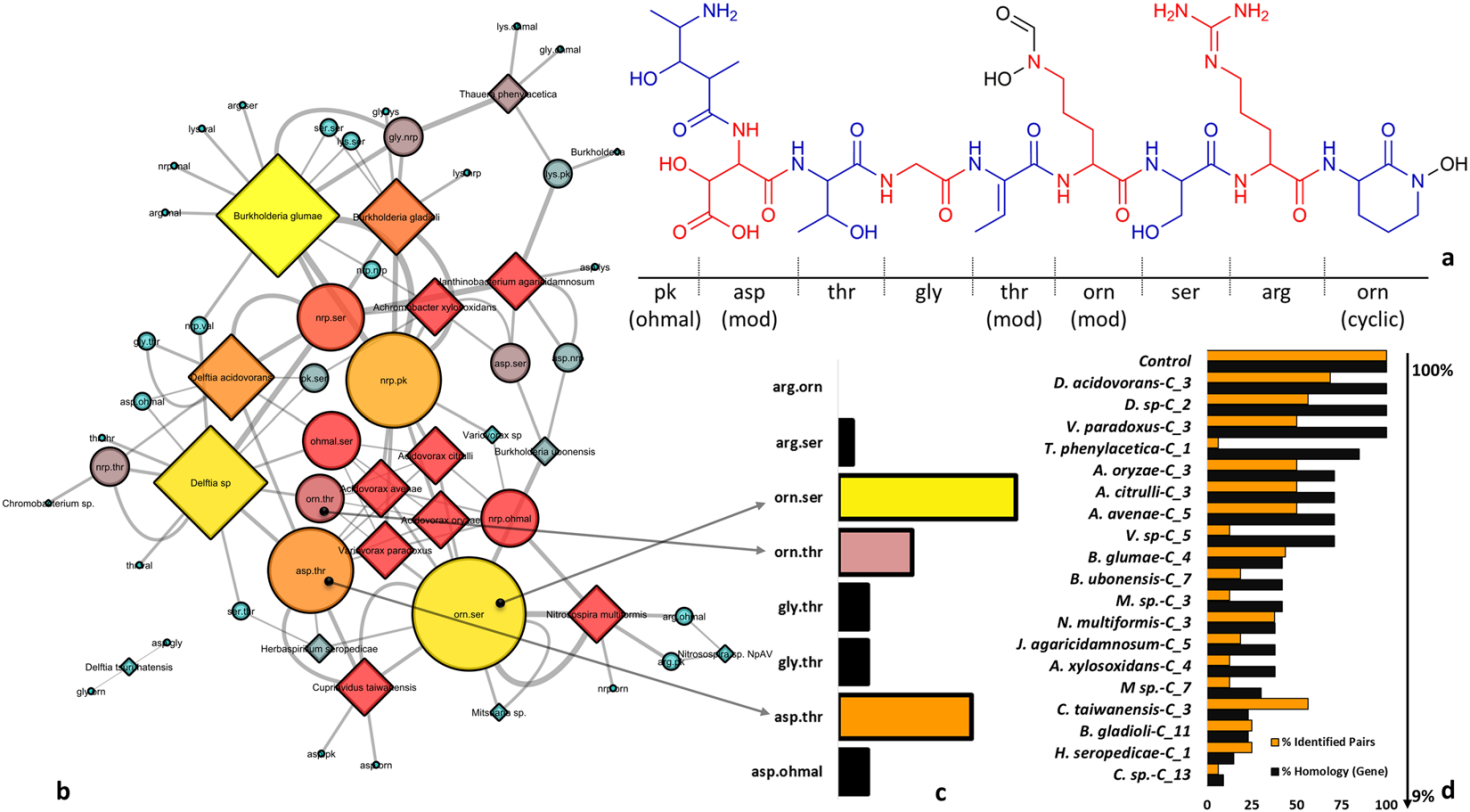
Species of the class BPB that exhibited BGCs associated with the biosynthesis of Delftibactin. (**a**) The structure of Delftibactin highlighting each subunit and the posttranslational modification sites (*mod* = modified and *cyclic* = cyclized form of the original peptide). (**b**) POPs associated to Delftibactin BGCs distributed in the dynamic network. (**c**) Percentage of each POP (matched with the original structure) based on PECs of Delftibactin. (**d**) Correlation between genomic identity of BGCs (black) and percentage of correct POPs (orange). Colors and sizes of nodes are proportional to the number of directed edges of each variable in the network. The diamond shape represents microorganisms and the circle shape represents POPs. Nodes sizes are proportional to the incidence of each variable. The Delftibactin dataset which describe the system of points is available at Supplementary Session as Supplementary File 1, named Delftibactin_validation).

To validate the second step of our protocol (POPs vs. PECs) the structure of the nonribosomal peptide Delftibactin, a metallophore associated to gold biomineralization^40^, was utilized as template. Its structure contains a classic hybrid pathway product of NRPS-PKS, and its peptide backbone is composed by the following peptides, in which some are associated with modifications of the original building blocks: ($p{k}_{(N{H}_{2}+ohmal)}$ + *Asp* + *Thr* + *Gly* + *Thr*_(*mod*)_ + *Orn*_(*mod*)_ + *Ser* + *Arg* + *Orn*_(*cyclic*)_) (Fig. 3a). The fragmentation of Delftibactin afforded Delftibactin PECs asp.pk, asp.thr, gly.thr, orn.thr, orn.ser, arg.ser, and arg.orn. In addition, gene clusters associated with the production of Delftibactin had their predicted core structures fragmented *two-by-two*, creating POPs. This analysis utilized the level of genomic identity, obtained from antiSMASH results, among BGCs for the biosynthesis of Delftibactin. Thus, PECs and POPs were compared searching for equivalent subunits.

The products of Delftibactin gene clusters (MIBiG: BGC0000984) were demonstrated as a dynamic network (Fig. 3b). The percentage of identity among Delftibactin gene clusters varied from 9 to 100%. The most commons POPs encountered were orn.ser (10.9%), asp.thr (8.18%), orn.thr (4.55%), in accordance with the peptide backbone of Delftibactin. The product of a hybrid NRPS system, the POP asp.ohmal, presented lower occurrence than other POPs (1.82%) (Fig. 3c). The POP gly.thr, which occurs twice in the structure of Delftibactin corresponded to 3.64% of the results. Moreover, PECs and POPs of Delftibactin matched correctly when their BGCs of origin exhibited higher levels of genomic identity. This characteristic is directly proportional, since the decreasing in the levels of identity led to divergent POPs to those of Delftibactin structure, eg. meaningless POPs to the identification of biosynthetic patterns. In other words, only genomic sequences presenting high percentage of identity contributed to the analysis with correct matches. The development of a simple system of points allowed this interpretation. The sum of points reached by each subunit permitted the recognition of structural similarities between PECs, POPs (Fig. 3d). The presence of outliers were also identified, in which the gene cluster 3 of *Variovorax paradoxus*, predicted with high level of similarity with Delftibactin gene cluster, resulted in a small percentage of identified pairs (Fig. 3d). However, the great majority of gene clusters pointed to similar results. The gene cluster 3 of *V. paradoxus* pointed to the following POPs: nrp.pk (0.5 points), ohmal.nrp (0.5 points), ohmal.ser (0 points), orn.ser (1 point), orn.thr (1 point), and asp.thr (1 point). Subunits reaching 1 point indicate that POPs and PECs perfectly matched. Even with divergent POPs, this gene cluster resulted in the most expressed subunits for delftibactin (orn.ser, orn.thr, and asp.thr). This fact supports the evidence that gene clusters of closely related species result in a certain level of similarity in the biosynthesis of subunits of an entire molecule. Thus, the analysis of Delftibactin gene clusters using RINPEP strategy pointed to the identification of POPs often expressed and what species are associated to this biosynthetic pattern.

These two-step experiments, comprised of a) PECs *vs*. POPs (Fig. 2) and b) POPs *vs*. PECs (Fig. 3), were consistent, revealing that fragments from antiSMASH predictions and NPs associated with their gene clusters exhibit patterns in the biosynthesis of NPs.

## Discussion

The RINPEP dataset resulted from the association of BGCs of different species and their respective products rearranged by pairs of monomers (PECs or POPs). The identification of *n* numbers of gene clusters pointing to similar products resulted in possible patters in the biosynthesis. The products of similar genomes lead to the creation of a dataset that allowed the identification of sites with small chances of modifications in the biosynthesis of nonribosomal peptides by species of the class BPB. In addition, similar POPs obtained from different genomes enforce that a specific part of the molecule has a major probability of being biosynthesized. It does not imply that under chemical stress or other conditions, the molecule will present the same peptide backbone, differently of the POPs that probably still occur. This is an event linked to elicitors of silent gene clusters that can, under different stimulation, generate different responses, in this case, a new metabolite^41^.

**Figure 4:**
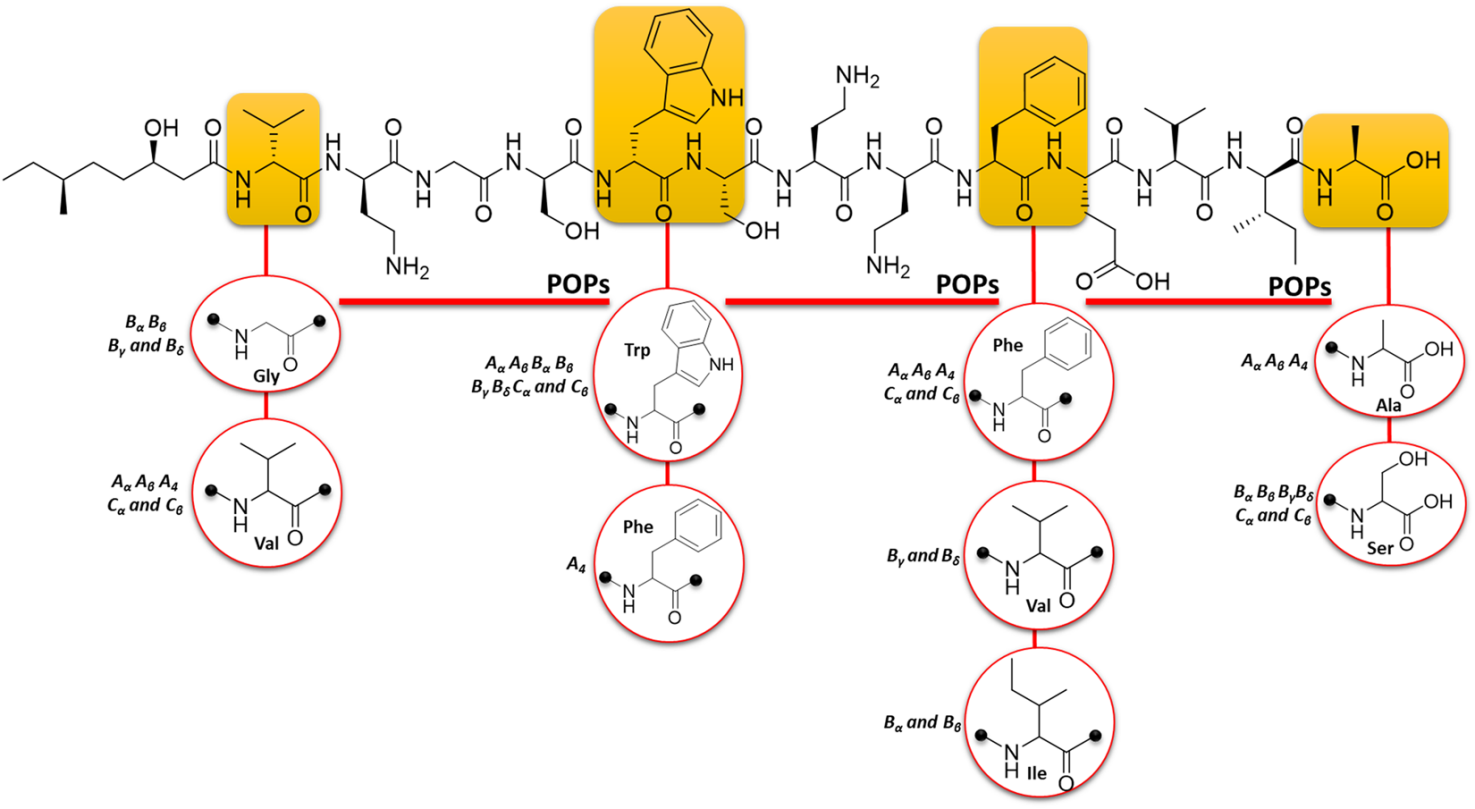
The structure of Tridecaptin biosynthesized by *Bacillus sp*. This image explains the substrate flexibility pointing to similar products among closely related species. The sites of substrate flexibility in yellow exhibit the respective amino acid linked to the other monomers encountered in different tridecaptins. The red lines below some peptides indicate that these amino acids do not undergo variations in the biosynthesis of tridecaptins from different species of *Bacillus sp*. These amino acids are the possible POPs for tridecaptin as an illustration of the RINPEP strategy for BPB species.

The recognition of patterns in POPs presented by *n* species increase the chance of finding fragments without discarding the ability of these species to add different subunits beyond the POP recognized as pattern. In this way, we reorganized the information extracted from antiSMASH and proposed here that if some molecular features could be identified as common in similar genomes this is an indicative of biosynthetic similaritiy. The Fig. 4 illustrates this event using one of the several examples in the field of NPs represented by tridecaptins, produced by several species of the genus *Bacillus sp*. There are nine tridecaptins named tridecaptin A_α_, A_β_, A_4_, B_α_, B_β_, B_γ_, B_δ_, C_α_, and C_β_^42^. These nonribosomal peptides present eight amino acids that are the same in all the nine tridecaptins. However, five of them are different in their structures. The eight amino acids that do not undergo modifications could be organized as POPs in an equivalent manner as proposed by RINPEP. In other words, these amino acids are similar in all tridecaptins implying in biosynthetic similarities by closely related species.

**Figure 5:**
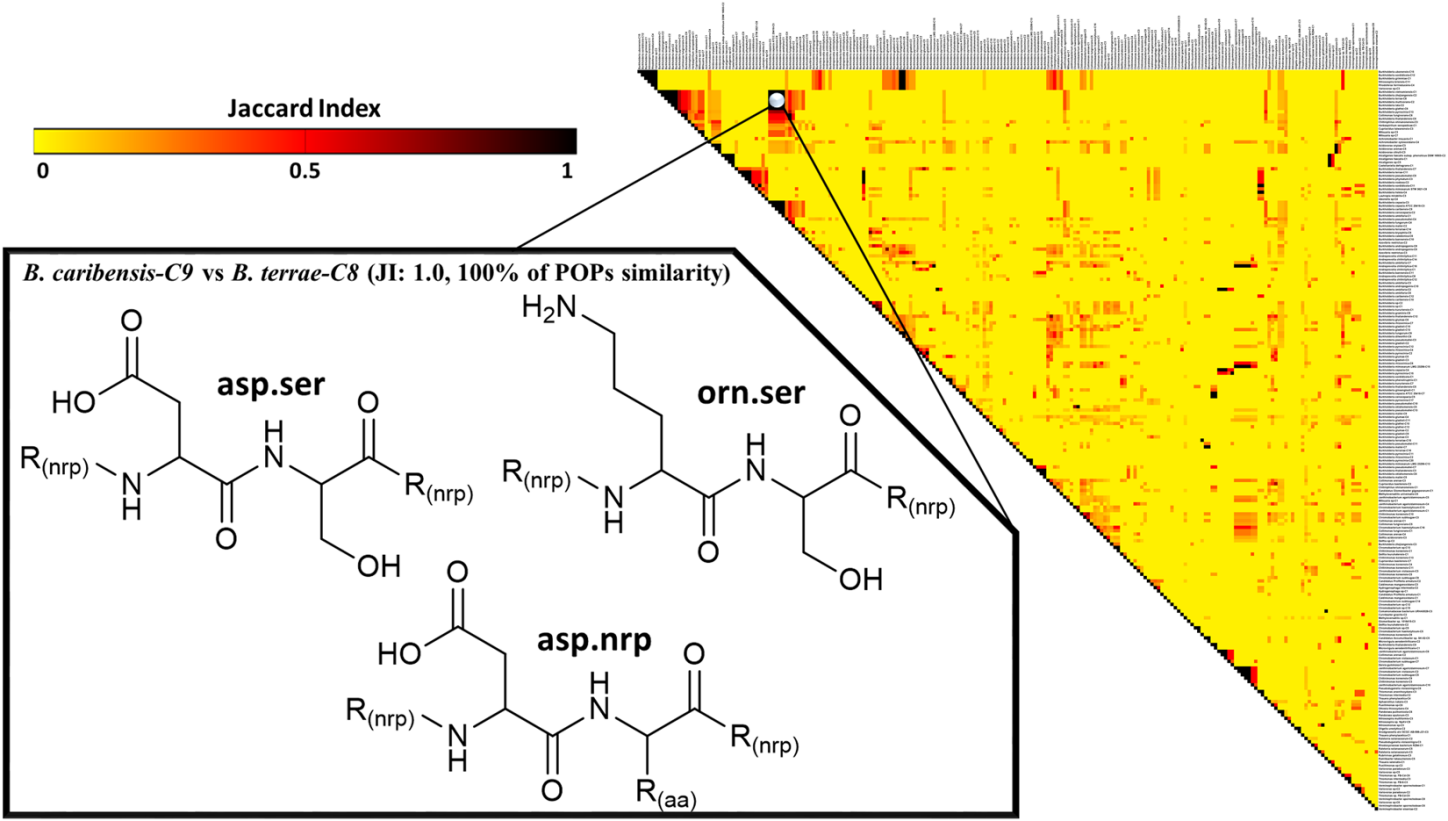
The heatmap built according to POPs from each gene cluster identified in BPB species. The highlighted region represents one of the clusters containing species with elevated values of JI. Values of JI closer to dark red and black mean that POPs are convergent among different species. Values of JI closer to yellow represent clusters which do not exhibit similarities between POPs. The heatmap was built in Gitools 2.2.2 overlapping POPs and their respective species using Jaccard Index. The hierarchical method based on Euclidean distance for multivariate analysis was applied for clustering variables with similar POPs.

Thus, RINPEP found sites with no modification in the biosynthesis of nonribosomal peptides in a product-centered approach. The dataset was organized and submitted to a visualization tool, which is able to create values for comparing POPs of these species. These similarities values were obtained via JI, represented as a heatmap. This analysis revealed several gene clusters responsible for clustering species based on similarities of POPs. The reason of employing heatmaps for showing these results is due to its advantageous manner of compressing a huge amount of data in an organized and logic representation. In addition, JI values are essentially correlated with a natural cutoff of variables, in which small JI values result in low occurrence of similar variables. As an example, it was noticed that *B. caribensis-C9* and *B. terrae-C8* (JI = 1.0) exhibit same POPs (asp.nrp, asp.ser, orn.ser). Thus, regions with higher JI permitted to infer about equivalent POPs and the respective gene cluster (Fig. 5).

The values of JI are normalized from zero to one, wherein higher values are associated to elevated similarity of variables. The heatmap shows these results as a whole and the existence of several gene clusters is the most important feature for the comparisons. Consequently, variables with elevated JI summarize equivalent POPs from similar gene clusters. The POPs behind the JI values are associated with biosynthetic patterns. In this sense, the values of JI must be interpreted in two manners: (**a**) higher JI values suggest the existence of patterns (as in the case of *B. caribensis-C9* and *B. terrae-C8*); and (**b**) lower values point to divergent POPs (as in the case of *V. paradoxus-C2* and *B. ambifaria-C1*, JI = 0.0). The dataset containing the values of JI for POPs is available at the Supplementary Session as Supplementary File S1, named Matrix_JI_Values.

The RINPEP strategy was created based on analogous NPs biosynthesized by closely related species^31^. The POPs obtained from gene clusters is a simplified manner of finding biosynthetic patterns in a product-centered overview.

A test dataset based on monomers (one peptide alone) and triads (three peptides linked) were also used with the purpose of finding the most appropriate mode of retrieving useful information from RINPEP. The results obtained for monomers did not provide any functional information. However, the same strategy proceeded by triads was ineffective due to the small number of associations, diminishing the number of species participating in the heatmap. Thus, the pairs design shows itself a more appropriate approach for the purpose of this study, since increasing the number of monomers decreases the chance of hitting a particular subunit. Heatmaps for monomers and triads of peptides are available at the Supplementary Session as Supplementary Figure 2.

The biosynthesis of similar portions of a molecule, or even the entire peptide backbone by NRPS of closely related species was previously noticed in some NRPS-PKS systems which produce siderophores^5, 6, 31, 43^. This suggests that this information can be used in a chemosystematic point of view^31^. As in the case of Delftibactin gene clusters, the selection of similar genomes is a key point for the identification of biosynthetic patterns.

**Figure 6:**
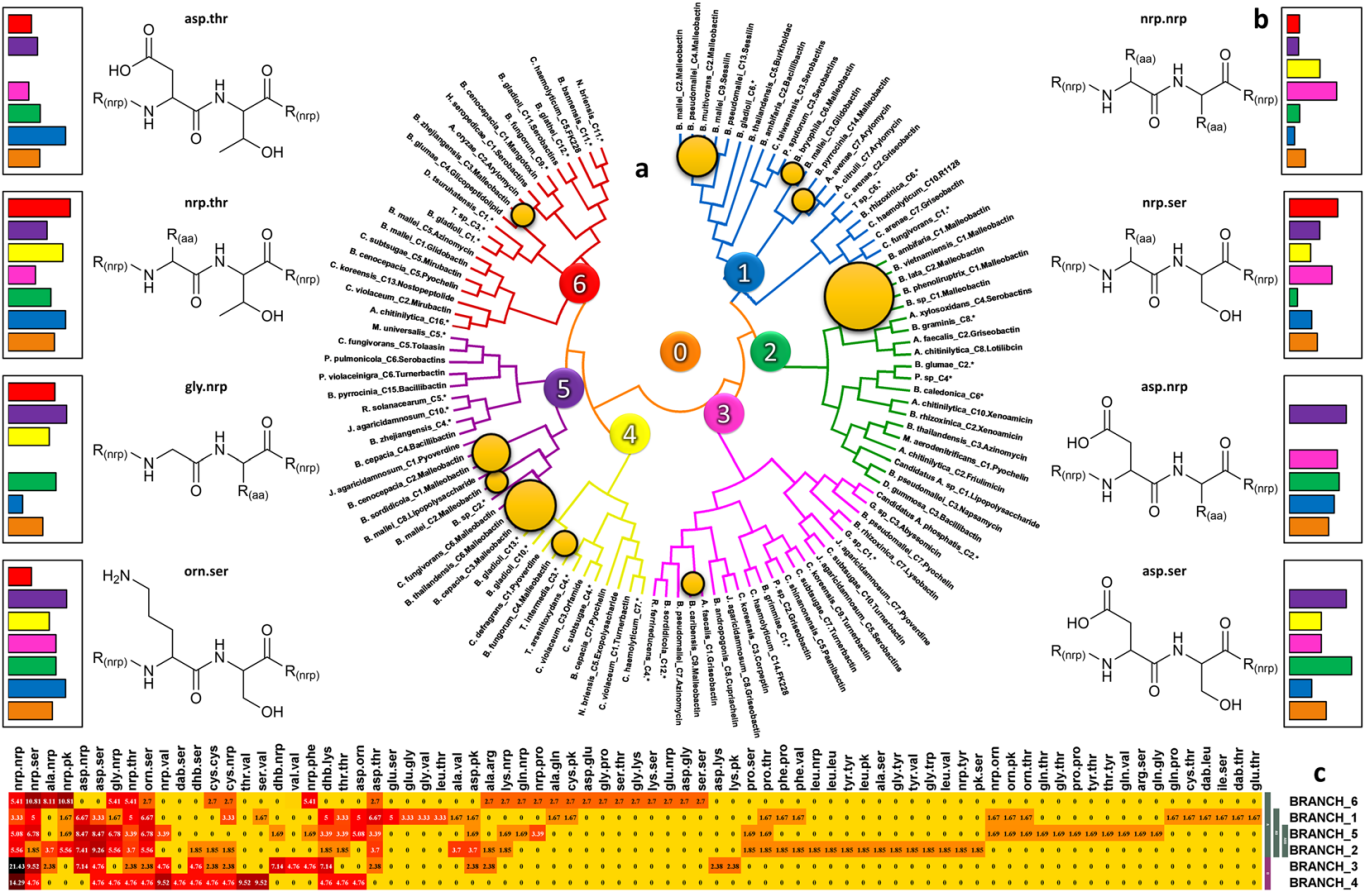
The dendrogram aligned by MEGA 6,  and the heatmap exhibiting the distribution of POPs in the main branches. The dendrogram was divided in main branches from 1 to 6. (**a**) The number zero (orange) represents the general occurrence of POPs in the entire dendrogram also displayed in each bar graph. The yellow circles represent the occurrence of BGCs associated with Malleobactin, which are proportional to the number of clusters. (**b**) Each bar graph is associated with its respective POPs and the bars represent its percentage of occurrence. The bar colors are associated with each main division in the dendrogram. The absence of bars means absence of POPs. The bar graphs were built in Excel. The gene cluster sequences were aligned by Mega 6 with the algorithm Clustal W, using the Neighbor-Joining method. (**c**) The heatmap showing the incidence of each POP in each main branch. The heatmap was created by Gitools v.2.2.2 based on POPs distribution. *BGCs that do not have their sequences associated to known NPs.

In this context, gene clusters sequences of 113 BGCs were downloaded, aligned, and deposited in a dendrogram with the purpose of investigating the distribution of POPs (Fig. 6a). The dendrogram exhibited different ranges of similarities between Malleobactin gene clusters (MIBiG: BGC0000386) from 14% to 92%, involving species from the genus *Burkholderia* (15 species) and *Collimonas* (1 species). These sixteen gene clusters exhibited POPs as asp.ser (22.22%); orn.ser (20.37%); asp.nrp (14.81%); and asp.orn (7.41%). However, the occurrence of divergent POPs to those of Malleobactin was noticed only in gene clusters with low similarity, corroborating results of JI values. In both cases, low genomic identity led to divergent POPs and high genomic identity led to correct matches as observed for Delftibactin. This observation confirms the influence of high genomic identity for the level of similarity in final products.

Malleobactin gene clusters with 92% of genomic identity showed only POPs present its structure. POPs from gene clusters with low level of similarity as well as, thr.thr; gly.nrp; asp.gly; asp.glu; arg.ser, were identified. A detailed information of Malleobactin POPs and gene clusters identity is available at the Supplementary session as Supplementary Figure 3.

The POP nrp.nrp, which do not provide any significant data about the type of peptide, indicates the existence of NRPS domains. A relative high percentage of nrp.nrp was observed in Branch_3 and Branch_4, with 21.43% and 14.29%, respectively, suggesting that their gene clusters do not resulted in the identification of the correct monomer.

The heatmap of Fig. 5 (section C) pointed to the existence of POPs which are restricted to some branches, such as gln.pro, cys.thr, dab.leu, ile.ser, dab.thr, and glu.thr, only present in the Branch_1. However, the distribution of asp.ser and orn.ser is global (Fig. 6b). In this sense, the wide occurrence of asp.ser and orn.ser and the elevated values of JI associated to species which present them, these POPs could be an indicative of biosynthetic patterns in species of BPB. As an example, species of the genus *Burkholderia* are associated to the production of NPs with the subunits orn.ser and asp.ser as portions of the entire molecule^44^.

In addition, genomic sequences associated with known compounds, such as Malleobactin, Serobactins and Sesillins were frequently grouped neighboring each other in the dendrogram, indicating genomic similarity. The heatmaps of POPs also suggest that the distribution of gene clusters from hybrid pathways is common in the species of the class BPB (POPs as cys.pk; pk.ser; dab.leu, for example), due to their common occurrence in some branches. Detailed information of the dendrogram dataset is available at the Supplementary Session as Supplementary File 1, named Dendrogram_Data and Dendrogram_POPs).

The RINPEP strategy was created to identify biosynthetic patterns in closely related species facilitating chemical investigation of species of the class BPB. Thus, the dataset revealed subunits that possibly do not undergo modifications in the biosynthesis of nonribosomal peptides as a fingerprint for these gene clusters.

## Conclusion

The strategy proposed by RINPEP revealed patterns in the biosynthesis of nonribosomal peptides based on molecular subunits. The genomic analysis carried out by antiSMASH and the data obtained from NCBI populated the data for this analysis. The RINPEP dataset comprises the products of BGCs based on their similarities calculated by Jaccard Index, in which different levels of genomic identity were recognized to carry important information for the identification of biosynthetic patterns. In this sense, by increasing the number of genomic sequences pointing to similar products its practical evidence is also enforced. Thus, the investigation of POPs and their association with gene clusters is a simple manner of finding biosynthetic fingerprints in a product-centered way, since higher values of JI point to a portion of a complex structure that is often present. Finally, the extrapolation of RINPEP for other classes of microorganism is feasible following the same purpose as suggested in this study.

## Methods

### Datasets

Genomic sequences from species of the class Betaproteobacteria were downloaded from the National Center of Biotechnology Information genome database (http://www.ncbi.nlm.nih.gov/genome). A complete list of species is available at the Supplementary Session as Supplementary File 1, named Microorganisms_List. Partially complete genomes were downloaded according to their most complete sequences. The sequences downloaded in this investigation were based in complete and draft genomes, since the objective is to use their expression, eg. the final predicted natural product. The dataset was created by merging results obtained from antiSMASH^45, 46^ and NCBI database containing basic information such as, microorganism name and taxonomy, genome size, %GC, gene counting, number of gene clusters, location, type, most similar known gene cluster, and prediction details.

### Biosynthetic gene cluster finder and predicted core structures

The identification of BGCs and details of predictions were carried out using antiSMASH. The results obtained for genomic sequences correlated with NRPS pathway consisted in detailed functional domain annotation, predicted core structure, and levels of genomic identity to known BGCs catalogued in MIBiG (http://mibig.secondarymetabolites.org)^38^. The predicted core structures were used according to the general consensus between NRPSprediction2, Stachelhaus code and Minowa, in which only matching predictions among them allowed the predicted peptide to be incorporated into the dataset. The incongruence between them (while each method generate different subunits for a domain) was considered as nrp (meaning that the R group could not be correctly assigned).

### The fragmentation of structures (*in silico*) and the creation of PECs and POPs

The analysis carried out in antiSMASH provided two important sources of data for RINPEP: a) the predicted core structures and b) the existent compound from the gene cluster with higher level of similarity from MIBiG database^38^. The structures of both, predicted and existent compounds, were used for populating the RINPEP dataset. Structures resulted from predictions and most similar known gene clusters (existent compounds) were fragmented *in silico* and unified in pairs of monomers. These fragments, grouped *two-by-two* created Pairs of Existent Compounds (PECs) and Pairs of Predictions (POPs), respectively. Each variable (POPs or PECs) was linked to the source of information eg. genome sequence, gene cluster number, and type of natural product. The structures of existent compounds indicated by antiSMASH results were obtained from the literature or compound databases as Chemspider (http://www.chemspider.com) or PubChem (https://pubchem.ncbi.nlm.nih.gov) and arranged for fitting in the RINPEP strategy.

POPs and PECs were normalized in alphabetical order (for example, a nonribosomal peptide containing four peptides, eg. ala.val.orn.ser, was transformed in the following pairs ala.val; orn.val; and orn.ser. A chemical point of view was taken for this normalization because the normalized subunit will be identified indifferently of its ordering in a mass or NMR spectrum based on its mass or heteronuclear correlations, respectively. Thus, this statement implies directly on the product. Structures of POPs and PECs were submitted to bioinformatics tools and used for finding similarities between BPB species using a two-step experiment: a) PECs *vs*. POPs (levels of genomic identity *vs*. predictions) and b) POPs *vs*. PECs (correct predictions *vs*. a natural product). The files created in these analyses were utilized for the construction of dynamic networks, heatmaps and the interpretation of the dendrogram. As predicted core structures and existent compounds were fragmented *in silico*, two common structural abbreviations were adopted in this work: 1) “R_(nrp)_”: which represents the remainder of the peptide backbone chain; and 2) “R_(aa)_”: which represents the amino acid that conferred an out of consensus moiety (unrecognized peptide side chain from antiSMASH results). PECs and POPs were normalized according to antiSMASH, KEGG^47^, and Norine^48^ nomenclatures.

### The system of points for the verification of RINPEP efficacy

A simple system of points was created for comparing the percentage of similarity of an identified gene cluster (from investigated sequences) with the product of the most similar known gene cluster (from MIBiG). This system of points consisted in the identification of correct (1 point), partially correct (0.5 points), and incorrect matches (0 points) between POPs and PECs in a product-centered way. Each correct match between PECs and POPs (eg. NPs_(PECs)_: ala.val; Prediction_(POPs)_: ala.val) had 1 point (100% of correspondence); Partially correct matches, containing only one out of consensus subunit (eg. NPs_(PECs)_: cys.met; Prediction_(POPs)_: cys.nrp) reached 0.5 points (50% of correspondence between PECs and POPs). Incorrect matches (represented as nrp.nrp, or different portions of the original molecule) reached 0 points (0% of correspondence between PECs and POPs). The final summing of points for each predicted core structure was associated with the percentage of similarity with the most similar known gene cluster from MIBiG. These estimations were carried out using the software Excel, simply by creating bar graphs with the percentage of correct POPs and the level of similarity with the most known gene cluster simultaneously.

### Parameters for the construction of the dendrogram

Genomic sequences of each gene cluster associated to the NRPS pathway were downloaded from the database NCBI in accordance with antiSMASH results as “.fasta” format and named as their respective species and number of gene clusters. The genomic sequences were allocated in one general fasta file and submitted to alignment. The dendrogram was built in MEGA6^49^. The analysis involved 113 sequences aligned by CLUSTAL W using default parameters^50^. The distribution of BGCs was inferred using the Neighbor-Joining method^51^. The dendrogram was divided in six main branches and the distribution of POPs was calculated and exhibited in the form of a heatmap. The heatmap was clustered based on the results of the Euclidean distance algorithm.

### Heatmaps based on the values of Jaccard Index for the identification of similarities

The stand-alone software Gitools v.2.2.2^52^ was utilized to provide integrative and visual analyses of POPs and species of the class BPB. The analysis based on the creation of heatmaps using hierarchical methods were carried out with the purpose of using values of Jaccard Index for the identification of similarities among species of the class BPB and their POPs. The classification of similar species was based on values of JI where values closer to zero represent lower levels of similarity and values closer to 1, higher levels of similarity. The heatmap was constructed based on the main dataset. The dataset was organized by the association of microorganisms with their respective POPs. The settings of hierarchical clustering were measured with Euclidean distance algorithm and the similarities were linked by their average.

### Dynamic network analysis

The networks were built with the software Cytoscape 3.3.0. The dataset was organized according to the software requirements by linking directly microorganisms (sources) and their features (target). The algorithm Allegro Layout 2.2.3 with Fruchterman-Reingold Layout was utilized. The network was treated according to parameters of the network analyzer. The size and colors of nodes were based on degrees of interaction among species, BGCs, and homology to different compounds. The diamond shape is associated to microorganisms and the circle shape represents the compound and level of homology. Other specific statistical analyses of the network were performed using the software Excel.

## Electronic supplementary material

Supplementary File 1

Supplementary File 2

## References

[1] Amini S, Tavazoie S (2011). Antibiotics and the post-genome revolution. Curr. Opin. Microbiol..

[2] Newman DJ, Cragg GM (2012). Natural products as sources of new drugs over the 30 years from 1981 to 2010. J. Nat. Prod..

[3] Felnagle EA (2008). Nonribosomal peptide synthetases involved in the production of medically relevant natural products. Mol. Pharm..

[4] Schwarzer D, Finking R, Marahiel MA (2003). Nonribosomal peptides: from genes to products. Nat. Prod. Rep..

[5] Koehn FE, Carter GT (2005). The evolving role of natural products in drug discovery. Nat. Rev. Drug Discov..

[6] Challis GL, Ravel J, Townsend CA (2000). Predictive, structure-based model of amino acid recognition by nonribosomal peptide synthetase adenylation domains. Chem. Biol..

[7] Challis GL, Naismith JH (2004). Structural aspects of non-ribosomal peptide biosynthesis. Curr. Opin. Struct. Biol..

[8] Walsh CT, Gehring AM, Weinreb PH, Quadri LE, Flugel RS (1997). Post-translational modification of polyketide and nonribosomal peptide synthases. Curr. Opin. Chem. Biol..

[9] Prabakaran S, Lippens G, Steen H, Gunawardena J (2012). Post-translational modification: Nature’s escape from genetic imprisonment and the basis for dynamic information encoding. Wiley Interdiscip. Rev. Syst. Biol. Med..

[10] McIntosh JA, Donia MS, Schmidt EW (2009). Ribosomal peptide natural products: bridging the ribosomal and nonribosomal worlds. Nat. Prod. Rep..

[11] Seo J, Lee KJ (2004). Post-translational modifications and their biological functions: proteomic analysis and systematic approaches. J. Biochem. Mol. Biol..

[12] Walsh CT, Malcolmson SJ, Young TS (2012). Three ring posttranslational circuses: Insertion of oxazoles, thiazoles, and pyridines into protein-derived frameworks. ACS Chem. Biol..

[13] Harvey AL, Edrada-Ebel R, Quinn RJ (2015). The re-emergence of natural products for drug discovery in the genomics era. Nat. Rev. Drug Discov..

[14] Nikolouli K, Mossialos D (2012). Bioactive compounds synthesized by non-ribosomal peptide synthetases and type-I polyketide synthases discovered through genome-mining and metagenomics. Biotechnol. Lett..

[15] Medema MH, Fischbach MA (2015). Computational approaches to natural product discovery. Nat. Chem. Biol..

[16] Walsh, C. T. Insights into the chemical logic and enzymatic machinery of NRPS assembly lines. *Nat. Prod. Rep*. 127–135, doi:10.1039/c5np00035a (2016). 10.1039/c5np00035a 26175103

[17] Fischbach MA, Walsh CT (2006). Assembly-line enzymology for polyketide and nonribosomal peptide antibiotics: Logic machinery, and mechanisms. Chem. Rev..

[18] Keating TA, Walsh CT (1999). Initiation, elongation, and termination strategies in polyketide and polypeptide antibiotic biosynthesis. Curr. Opin. Chem. Biol..

[19] Rausch C, Weber T, Kohlbacher O, Wohlleben W, Huson DH (2005). Specificity prediction of adenylation domains in nonribosomal peptide synthetases (NRPS) using transductive support vector machines (TSVMs). Nucleic Acids Res..

[20] Schaffer ML, Otten LG (2009). Substrate flexibility of the adenylation reaction in the Tyrocidine non-ribosomal peptide synthetase. J. Mol. Catal. B Enzym..

[21] Xie Y (2014). NRPS substrate promiscuity leads to more potent antitubercular sansanmycin analogues. J. Nat. Prod..

[22] Crawford JM, Clardy J (2012). Microbial genome mining answers longstanding biosynthetic questions. Proc. Natl. Acad. Sci..

[23] Richards S (2015). It’s more than stamp collecting: How genome sequencing can unify biological research. Trends in Genetics.

[24] Cimermancic P (2014). Insights into Secondary Metabolism from a Global Analysis of Prokaryotic Biosynthetic Gene Clusters. Cell.

[25] Gaudêncio SP, Pereira F (2015). Dereplication: racing to speed up the natural products discovery process. Nat. Prod. Reports.

[26] Johnston CW (2015). An automated Genomes-to-Natural Products platform (GNP) for the discovery of modular natural products. Nat. Commun..

[27] Yang L (2015). Exploration of Nonribosomal Peptide Families with an Automated Informatic Search Algorithm. Chem. Biol..

[28] Ng J (2009). Dereplication and de novo sequencing of nonribosomal peptides. Nat Methods.

[29] Kersten RD (2011). A mass spectrometry–guided genome mining approach for natural product peptidogenomics. Nat. Chem. Biol..

[30] Mohimani H (2014). NRPquest: Coupling Mass Spectrometry and Genome Mining for Nonribosomal Peptide Discovery. J. Nat. Prod..

[31] Wang H, Fewer DP, Holm L, Rouhiainen L, Sivonen K (2014). Atlas of nonribosomal peptide and polyketide biosynthetic pathways reveals common occurrence of nonmodular enzymes. Proc. Natl. Acad. Sci..

[32] Forseth, R. R. *et al*. Homologous NRPS-like gene clusters mediate redundant small-molecule biosynthesis in Aspergillus flavus. *Angew. Chemie - Int. Ed*. **52**, 1590–1594 (2013). 10.1002/anie.201207456 PMC3758896 23281040

[33] Pidot SJ, Coyne S, Kloss F, Hertweck C (2014). Antibiotics from neglected bacterial sources. Int. J. Med. Microbiol..

[34] Ling LL (2015). A new antibiotic kills pathogens without detectable resistance. Nature.

[35] Weber T (2015). antiSMASH 3.0—a comprehensive resource for the genome mining of biosynthetic gene clusters. Nucleic Acids Res..

[36] Lassalle F (2015). GC-Content Evolution in Bacterial Genomes: The Biased Gene Conversion Hypothesis Expands. PLOS Genet..

[37] Foerstner KU, von Mering C, Hooper SD, Bork P (2005). Environments shape the nucleotide composition of genomes. EMBO Rep..

[38] Medema MH (2015). Minimum Information about a Biosynthetic Gene cluster. Nat. Chem. Biol..

[39] Walsh, C. T., O’Brien, R. V. & Khosla, C. Nonproteinogenic Amino Acid Building Blocks for Nonribosomal Peptide and Hybrid Polyketide Scaffolds. *Angew. Chemie Int. Ed*. **52**, 7098–7124 (2013). 10.1002/anie.201208344 PMC4634941 23729217

[40] Johnston CW (2013). Gold biomineralization by a metallophore from a gold-associated microbe. Nat. Chem. Biol..

[41] Seyedsayamdost MR (2014). High-throughput platform for the discovery of elicitors of silent bacterial gene clusters. Proc. Natl. Acad. Sci..

[42] Cochrane SA, Vederas JC (2016). Lipopeptides from Bacillus and Paenibacillus spp.: A Gold Mine of Antibiotic Candidates. Med. Res. Rev..

[43] Fisch KM (2013). Biosynthesis of natural products by microbial iterative hybrid PKS–NRPS. RSC Adv..

[44] Baldim JL, Soares MG (2016). An Insight Into the Intraspecific Variation of Biosynthetic Gene Clusters Between Strains of Burkholderia thailandensis spp. J. Braz. Chem. Soc..

[45] Medema MH (2011). antiSMASH: rapid identification, annotation and analysis of secondary metabolite biosynthesis gene clusters in bacterial and fungal genome sequences. Nucleic Acids Res..

[46] Blin K (2013). antiSMASH 2.0-A versatile platform for genome mining of secondary metabolite producers. Nucleic Acids Res..

[47] Kanehisa M, Goto S (2000). KEGG: Kyoto encyclopedia of genes and genomes. Nucleic Acids Res..

[48] Caboche S (2008). NORINE: A database of nonribosomal peptides. Nucleic Acids Res..

[49] Tamura K, Stecher G, Peterson D, Filipski A, Kumar S (2013). MEGA6: Molecular evolutionary genetics analysis version 6.0. Mol. Biol. Evol..

[50] Larkin MA (2007). Clustal W and Clustal X version 2. 0. Bioinformatics.

[51] Saitou N, Nei M (1987). The neighbor-joining nethod: A new method for reconstructing phylogenetic trees. Mol Biol Evol.

[52] Perez-Llamas C, Lopez-Bigas N (2011). Gitools: Analysis and visualisation of genomic data using interactive heat-maps. PLoS One.

