# Supplementary material for: A strategy for the identification of patterns in the biosynthesis of nonribosomal peptides by Betaproteobacteria species
Source: Sci Rep. 2017 Sep 4;7:10400. doi: 10.1038/s41598-017-11314-w (PMC5583390; doi:10.1038/s41598-017-11314-w)
Supplement: Supplementary file 2 [file 41598_2017_11314_MOESM2_ESM.pdf]

SUPPLEMENTARY SESSION

A strategy for the identification of patterns in the biosynthesis of nonribosomal peptides by Betaproteobacteria species

João Luiz Baldim[1,2*], Bruna Lidiane da Silva[1], Daniela Aparecida Chagas-Paula[1], João Henrique Ghilardi Lago[2], Marisi G. Soares[1]

[1]Institute of Chemistry, Federal University of Alfenas, Alfenas. 700, Gabriel Monteiro da Silva St. ZIPCODE: 37130-000 Alfenas, Minas Gerais – Brazil.
[2] Centre of Human and Natural Sciences - Federal University of ABC – UFABC, Santo André-SP, 09210-580, Brazil.
e-mail: jotaelebaldim@gmail.com

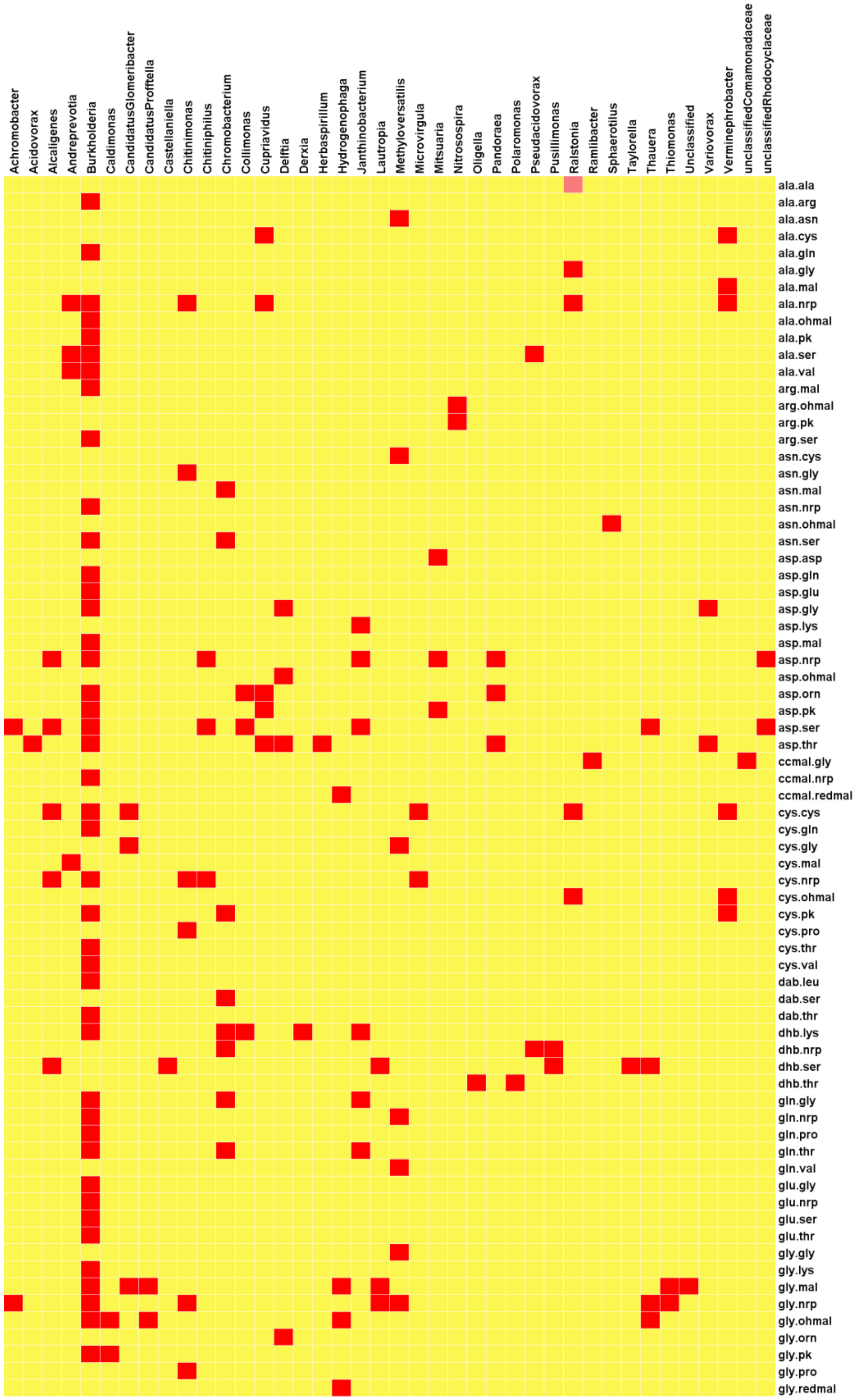

Supplementary Figure 1 (Part 1). The heatmap for the RINPEP dataset. The POPs occurrence were clustered according to their respective genus (POPS from ala.ala to gly.redmal).

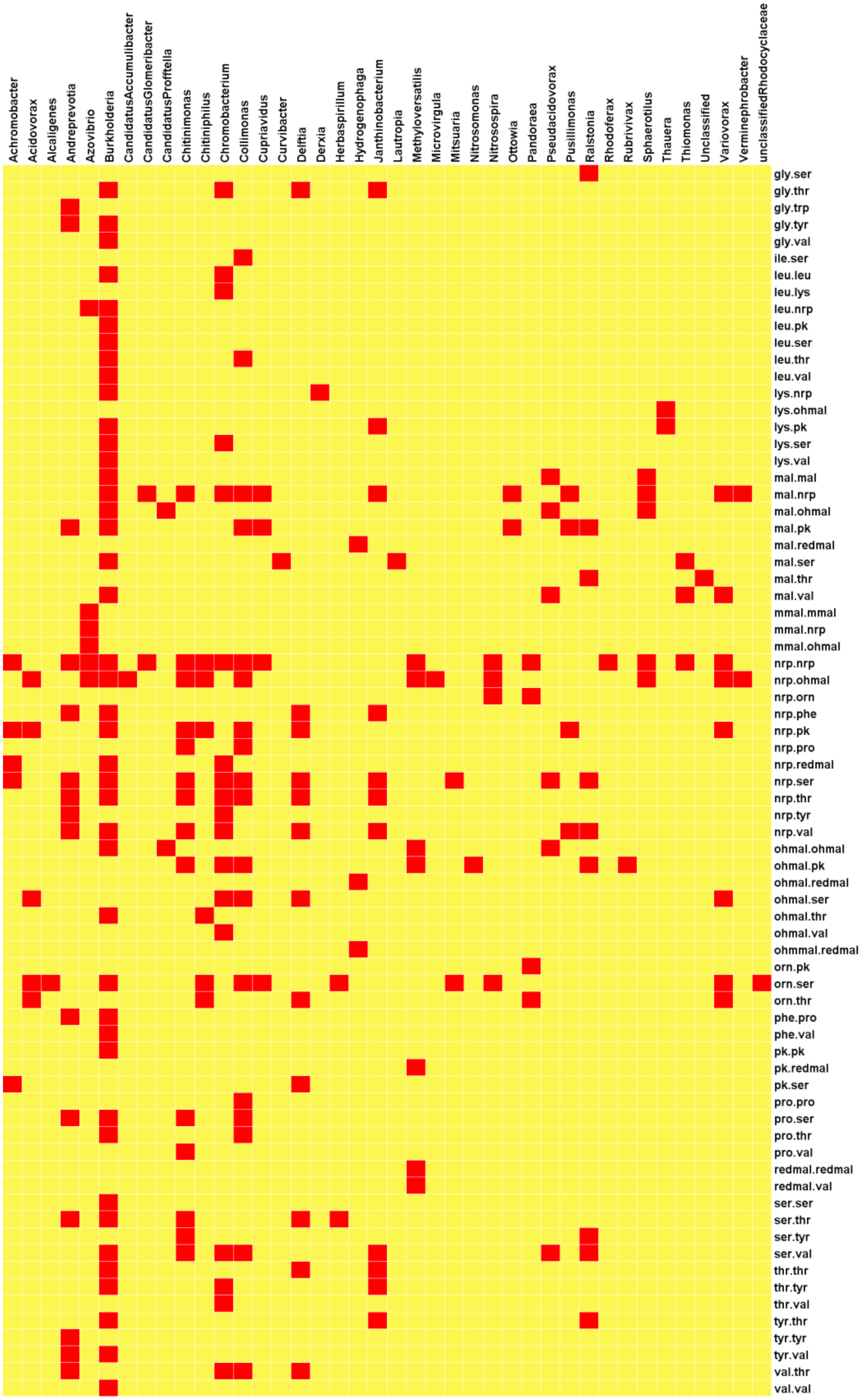

Supplementary Figure 1 (Part 2). The heatmap for the RINPEP dataset. The POPs occurrence were clustered according to their respective genus (POPs from gly.ser to val.val).

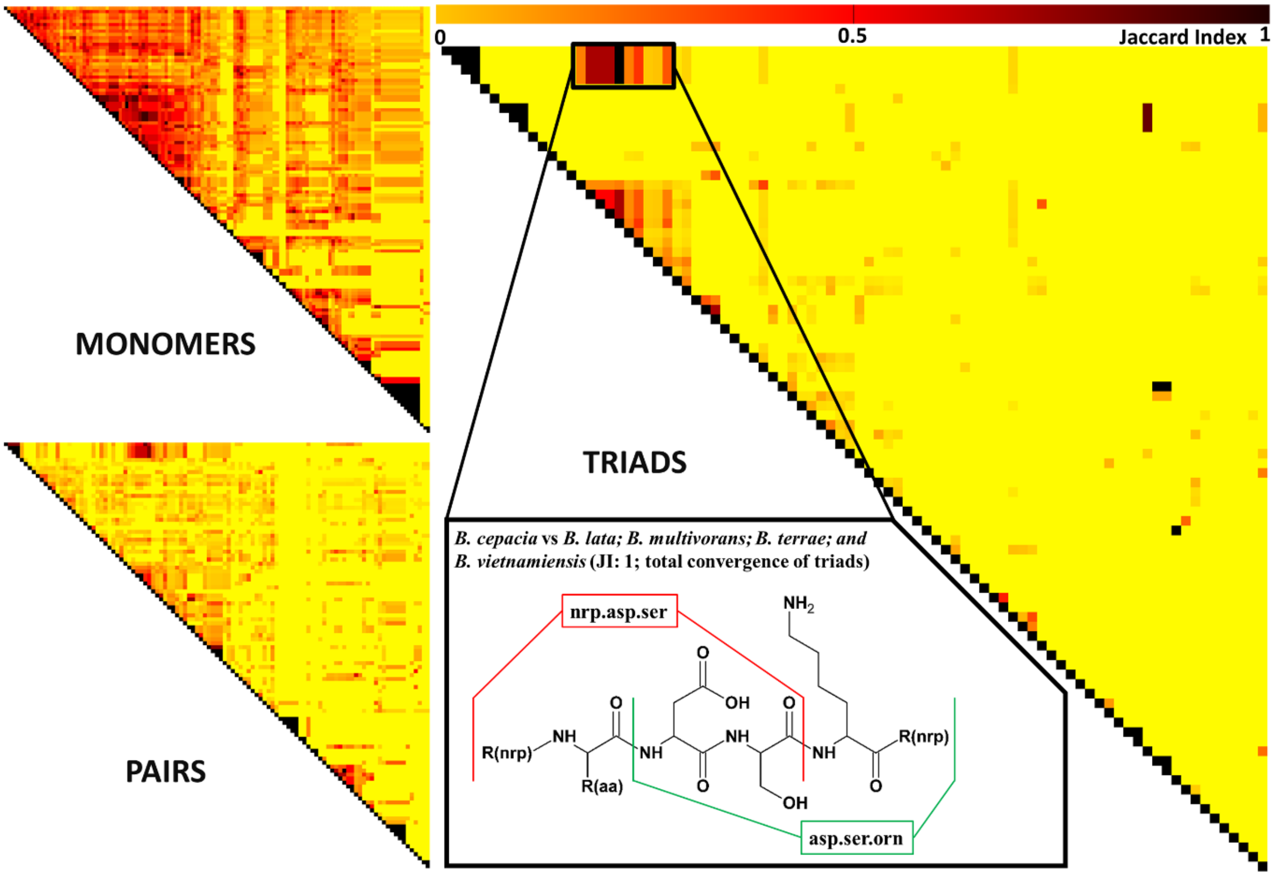

Supplementary Figure 2. Heatmaps for Monomers, Pairs and Triads, exhibiting values of JI. Values of JI closer to dark red and black mean that POPs are convergent between different species. Values of JI closer to yellow exhibit POPs which are divergent among species. The heatmap was built in Gitools 2.2.2 overlapping POPs and their respective species. The hierarchical method of multivariate analysis was applied to POPs.

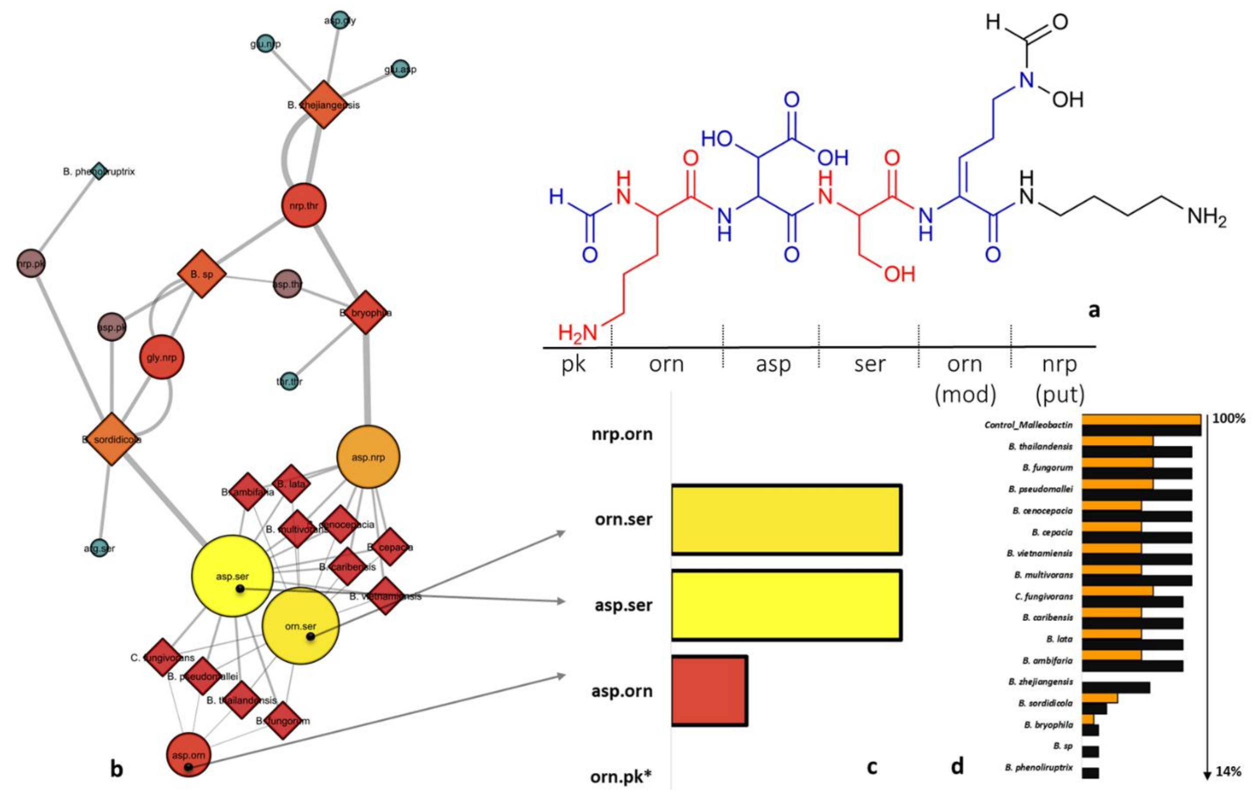

Supplementary Figure 3. Species of the class BPB that presented BGCs with different levels of genomic identity for the biosynthesis of Malleobactin. a) The structure of Malleobactin highlighting each subunit and the posttranslational modification sites (mod= modified and put= putrescine). b) POPs associated to Malleobactin BGCs. c) Percentage of each POP (matched with the original structure). d) Correlation between the genomic identity of BGCs (black) and the percentage of correct POPs (orange). Colors and sizes of nodes are proportional to the degree of each variable.
